# Total potentiation level: a new metric for quantifying post-activation potentiation dynamics using tensiomyography and statistical parametric mapping

**DOI:** 10.3389/fbioe.2025.1533749

**Published:** 2025-09-09

**Authors:** Srđan Đorđević, Elijan Mastnak, Jan Žumer, Simon Krašna, Maja Ćirić, Milivoj Dopsaj

**Affiliations:** ^1^ Research and Development Department, TMG-BMC, Ljubljana, Slovenia; ^2^ University of Ljubljana, Faculty of Mechanical Engineering, Ljubljana, Slovenia; ^3^ University of Belgrade, Faculty of Medicine, Belgrade, Serbia; ^4^ Department of Motor Skills and Methodology, University of Belgrade Faculty of Sport and Physical Education, Belgrade, Serbia

**Keywords:** post-activation potentiation, total potentiation level, tensiomyography, statistical parametric mapping, twitch potentiation, neuromuscular performance, muscle contractility

## Abstract

**Introduction:**

Post-activation potentiation (PAP), a transient increase in muscle twitch force after conditioning stimuli, may influence performance. Traditional discrete metrics often fail to resolve PAP’s time-dependent contractility changes. We introduce Total Potentiation Level (TPL)—the integrated area of significant potentiation over time derived from statistical parametric mapping (SPM) of tensiomyography (TMG) data in the rectus femoris—to quantify PAP holistically. We hypothesized that this romisingSPM-based TPL approach would more sensitively capture PAP’s temporal dynamics than traditional discrete measures.

**Methods:**

Fifty-eight physically active adults (36M/22F; 28.4 ± 11.0 years; normalized knee torque 1.395 ± 0.158Nm/kg) performed four sets of eight incline squats (ISQ) with individualized loads (10RM-based) and 150-s inter-set rest. TMG assessed rectus femoris twitch responses pre/post-ISQ. SPM analyzed potentiation profiles, with TPL derived from supra-threshold SPM t-continuum. TPL’s sensitivity to PAP dynamics was compared to traditional discrete metrics.

**Results:**

SPM analysis indicated that potentiation was maximized following the second ISQ set (TPL = 636.5; p < 0.0001), then plateaued with a slight decline by the fourth set. Statistically significant temporal changes in PAP were observed between 11.3 m and 62.6 m, a detail not discernible through conventional discrete measures. This suggests that TPL may offer enhanced sensitivity in identifying peak potentiation and early fatigue onset.

**Discussion:**

The findings suggest that TMG combined with SPM provides an approach for PAP quantification, with TPL potentially offering a comprehensive view of potentiation dynamics. TPL captures nuanced, continuous temporal changes not readily apparent in traditional discrete analyses and may inform more precise conditioning strategies. Further research is warranted to confirm these preliminary observations and explore broader applications.

**Conclusion:**

We developed TPL by combining tensiomyography TMG and statistical parametric mapping SPM. TPL uses SPM’s dynamic time-amplitude analysis to detect subtle, transient PAP shifts, enabling precise neuromuscular adaptation quantification. Its applications may span training, rehabilitation, and aging-related interventions by potentially optimizing conditioning parameters to enhance muscle contractility with minimal fatigue. TPL could also help identify optimal individualized loads to maximize contractile performance, with potential benefits for athletic and therapeutic outcomes and load management. Further studies are needed to validate TPL across various exercise modalities and populations, thereby increasing its applicability to tailored applications.

## 1 Introduction

Muscle contraction—encompassing both concentric (shortening) and eccentric (lengthening) actions—is fundamental to human movement and functional performance (Padulo et al., 2013), with acute and chronic changes in contractility modulated by competing phenomena: potentiation and fatigue (Allen et al., 2008; MacIntosh and Rassier, 2002a). PAP is a transient enhancement in muscle twitch force and rate of force development following high-intensity conditioning contractions (Blazevich and Babault, 2019; Jubeau et al., 2010). It contrasts with fatigue—a reduction in contractile capacity due to metabolic byproduct accumulation and impaired excitation-contraction coupling (Gandevia, 2001; Enoka and Duchateau, 2016; Debold et al., 2016). Voluntary exercise also induces fatigue, which affects the neuromuscular system’s central and peripheral components (Gandevia, 2001; Enoka and Duchateau, 2016; MacIntosh and Rassier, 2002b). Mechanistically, PAP is driven by the phosphorylation of myosin regulatory light chains, which increases actin-myosin cross-bridge sensitivity to calcium (Tillin and Bishop, 2009; Prieske et al., 2020) and increases the excitability of motor neurons (Blazevich and Babault, 2019; Prieske et al., 2020; Tillin and Bishop, 2009). This transient enhancement holds significant implications for optimizing explosive movements in sports, rehabilitation, and aging populations (Seitz and Haff, 2016; Hodgson et al., 2005). However, its quantification remains methodologically challenging, limiting its practical application. In this study, PAP is quantified *via* twitch potentiation (TP) measured through TMG in the rectus femoris muscle, providing direct insight into the muscle’s adaptive response; notably, the PAP–TP relationship is crucial for optimizing movement efficiency and explosive power (Sasaki et al., 2012).

Historically, PAP has been assessed using discrete metrics such as peak twitch torque, measured *via* joint-level mechanical impulses under isometric conditions (Ramsey and Street, 1941; Tillin and Bishop, 2009). While these methods provide snapshots of muscle performance, they are constrained by joint-damping effects, an inability to isolate specific muscles, and a focus on singular time points rather than dynamic temporal profiles (Dahmane et al., 2001). Tensiomyography (TMG), a non-invasive technique that records radial displacement of the muscle belly in response to electrical stimulation, addresses these limitations by enabling direct, *in situ* evaluation of contractile properties (Dahmane et al., 2005). TMG has been successfully used in previous studies to detect changes in muscle contractility following conditioning activities, demonstrating its sensitivity in capturing subtle alterations in muscle function (Dahmane et al., 2005). Despite its advantages, conventional TMG analyses reduce complex twitch responses to isolated parameters—e.g., contraction time (Tc), maximal displacement (Dm), or rate of deformation (RDD_max_)—overlooking the continuous interplay between potentiation and fatigue (García-Manso et al., 2011; Hughes et al., 2022). This reductionist approach fails to resolve PAP’s time-dependent dynamics, masking critical potentiation onset, peak, and decay phases.

A persistent gap in the literature lies in the absence of methodologies that holistically quantify PAP’s temporal and amplitude dimensions. Current practices often conflate PAP with post-activation performance enhancement (PAPE), though their mechanisms and measurement frameworks differ fundamentally. PAP reflects intrinsic changes in muscle contractility, measurable *via* evoked twitch responses (Hodgson et al., 2005). In contrast, PAPE describes improvements in voluntary tasks (e.g., jumps, sprints) attributed to neuromuscular or systemic factors such as increased muscle temperature or arousal (Prieske et al., 2020). This distinction underscores the need for tools that isolate PAP’s physiological signature from confounding variables. Existing studies neglect the cumulative effects of conditioning stimuli, such as the balance between potentiation and fatigue across repeated sets—a critical factor for designing effective training protocols (Rassier and Macintosh, 2000). These phenomena are not mutually exclusive; potentiation and fatigue can coexist depending on conditioning intensity and volume, and their interplay is influenced by muscle fiber composition, rest intervals, and individual conditioning status, which together determine whether potentiation or fatigue predominates after a given stimulus (Tillin and Bishop, 2009).

We introduce and validate Total Potentiation Level (TPL), a novel metric derived from integrating tensiomyography and statistical parametric mapping (SPM) to address these limitations. SPM, a robust analytical framework developed initially for neuroimaging (Friston et al., 1995), evaluates continuous time-series data to identify statistically significant temporal regions of potentiation (Pataky, 2012; Robinson et al., 2015). Unlike traditional discrete metrics, TPL quantifies PAP by integrating the magnitude and duration of supra-threshold potentiation across the entire twitch response, i.e., it represents the area under the largest supra-threshold cluster of the SPM t-continuum, providing a two-dimensional (time–amplitude) characterization of neuromuscular adaptation. In the present analysis, one-dimensional paired SPM t-tests were utilized to compare pre-versus post-conditioning TMG twitch curves, enabling a time-domain capture of PAP’s full temporal dynamics induced by the conditioning protocol. This approach captures transient shifts in muscle contractility and identifies optimal conditioning intervals to maximize performance while mitigating fatigue.

This study aims to introduce and validate TPL as a comprehensive tool for assessing PAP dynamics, bridging the methodological gap in traditional analyses. By combining TMG’s high-resolution contractile data with SPM’s temporal sensitivity, we aim to (1) characterize the time course of PAP across multiple conditioning sets, (2) establish TPL’s superiority over conventional discrete metrics in detecting nuanced potentiation–fatigue interactions, and (3) demonstrate how the TPL metric could enable practitioners to individualize athletic and rehabilitation protocols by identifying conditioning parameters that maximize strength and speed adaptations (both of which require high levels of potentiation) while avoiding premature fatigue. This approach would advance evidence-based programming by replacing generic guidelines with temporally resolved, individualized potentiation profiles, optimizing the balance between performance enhancement and neuromuscular fatigue management. Ultimately, by characterizing potentiation continuously, our approach could offer a holistic framework for optimizing muscle performance across athletic training, rehabilitation, and age-related neuromuscular decline contexts.

TPL aims to reduce comparison bias by avoiding the pre-selection of discrete variables (such as Tc and Dm). These discrete metrics reflect only brief moments of observation and may not capture the full time course of muscle response changes. The primary hypothesis of this study was that TPL would provide a more sensitive measure of PAP dynamics than traditional discrete metrics. Specifically, we predicted that TPL, by integrating the entire supra-threshold twitch response, would detect a non-linear, peak-and-decline pattern across conditioning sets, reflecting the interplay of potentiation and fatigue—single-point discrete parameters would obscure a dynamic we hypothesized.

## 2 Materials and methods

### 2.1 Participants

The present study recruited 58 participants 36 males and 22 females) with an average ±standard deviation), age of 28.4 ± 11.0 years, height of 176.29 ± 20.95 cm, and weight of 71.53 ± 14.46 kg from a pool of healthy and physically active students, recreational athletes, and track and field athletes. The participants reported exercising at least three times per week for more than 30 min per session and engaging in various sports or activities, such as strength exercises, running, cycling, swimming, or soccer. All participants had a minimum of 6 years of training experience, including regular engagement in basic strength training exercises. Furthermore, the ISQ exercise, integral to the conditioning activity, was novel to over 90% of the participants. To be eligible for participation, participants had to be free of leg injuries for at least 6 months before starting the test protocol. The participants were randomly selected using a computer-generated random number list. The study was approved by the Ethics Committee of the University of Belgrade Faculty of Sport and Physical Education (02 No. 484–2) and conducted in compliance with the World Medical Association’s Declaration of Helsinki (2013), which outlines ethical principles for research involving human subjects. The participants received a detailed explanation of the study protocol, and informed consent was obtained from all subjects. Each subject completed and signed a form that described the study clearly and understandably. The study ensured that all personal and experimental data remained confidential, adhering to best data protection and privacy practices. Data handling procedures complied with current EU General Data Protection Regulation (GDPR) standards to guarantee the participants’ privacy and data integrity throughout the research.

### 2.2 Study design

**FIGURE 1:**
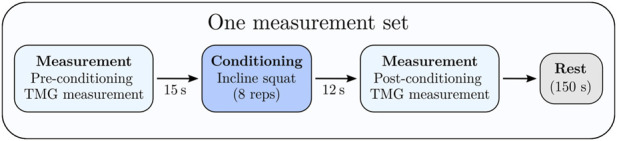
The study used one such set of conditioning exercises and measurement protocols. Each participant repeated one such measurement set four times for the testing protocol.

This study employed a repeated-measures design to examine acute changes in muscle contractile properties across a single exercise session. Participants performed four sets of eight repetitions of an incline squat exercise, with 150 s of inter-set rest. TMG measurements of the rectus femoris were taken immediately Pre and Post (less than 15 s after) each ISQ set, yielding eight measurement time points in total (Pre1, Post1, Pre2, Post2, … Pre4, Post4) throughout the session. The sequence of events in each trial was as follows: a baseline TMG measurement was obtained just before the first ISQ set, the participant then completed the 8-rep ISQ set, and a second TMG measurement was conducted immediately after the set. This Pre/Post measurement cycle was repeated for all four sets, with the post-set measurement conducted promptly (within ∼10–15 s) after the final repetition of each set and the remaining time used as rest before the next set. This design ensured that muscle twitch responses were captured both prior to and following the conditioning activity of each set, allowing assessment of fatigue and potentiation dynamics throughout the exercise bout Figure 1.

### 2.3 Conditioning exercise

The weighted incline squat (ISQ) was chosen as the conditioning exercise because the rectus femoris (RF) is activated to a very high level compared to other exercises where the hip angle is larger. A previous study by (Frigo et al., 2020) showed that the RF had an average activation level of 70% higher in low angles (0° compared to 80° hip angle). The high activation level of the RF is due to its bi-articular structure. A smaller hip angle means a longer RF length and a higher RF tension. Another reason for choosing ISQ as the conditioning exercise is that it is simple to perform requires no special equipment other than a ramp, and puts minimal stress on the back.

**FIGURE 2:**
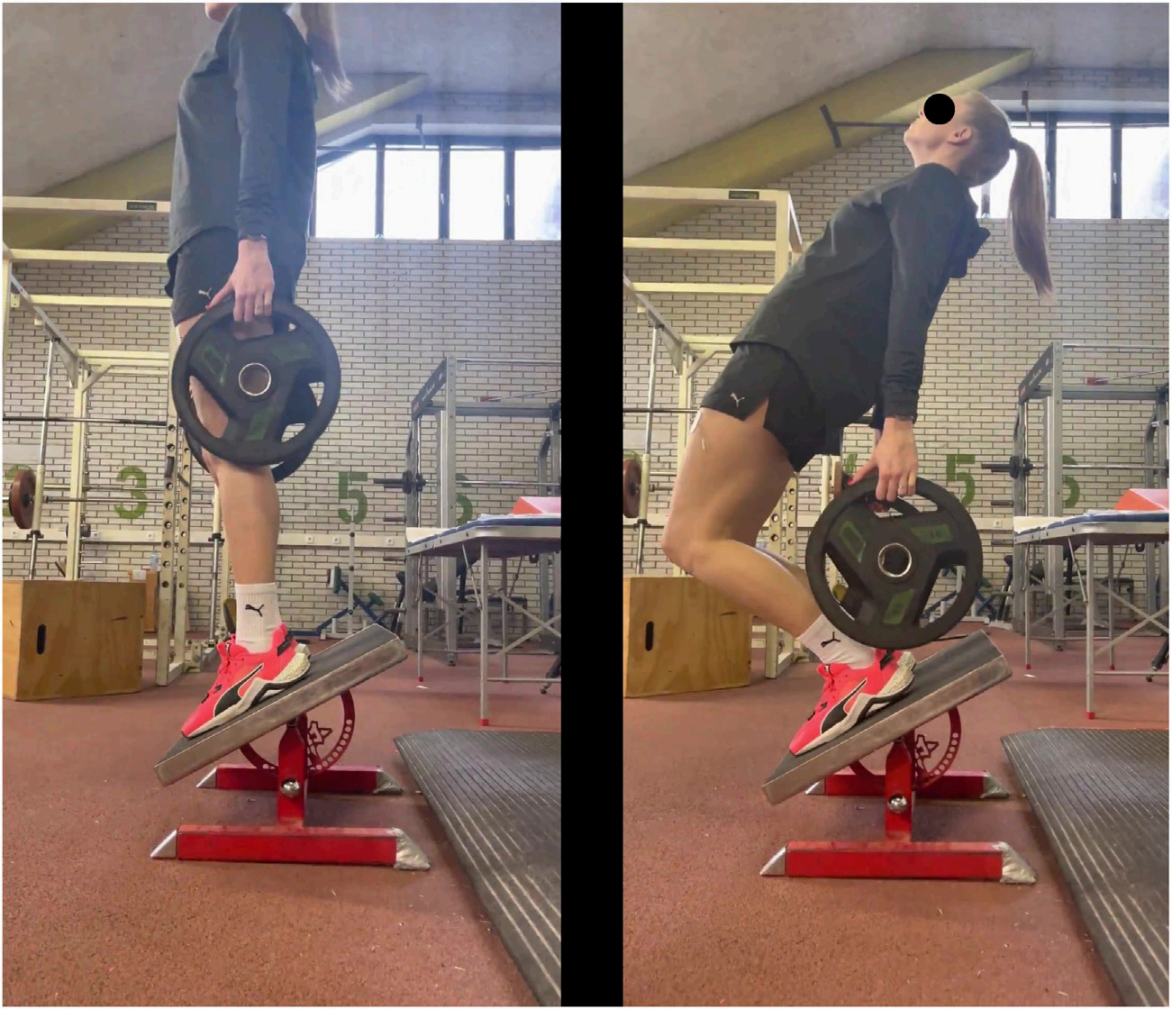
Incline squat: **(A)** down position, knee angle 0°; **(B)** up position, knee angle 90°.

The volunteers performed the ISQ on a platform with a slope angle of 30°, holding an additional load of between 2 × 2.5 kg and 2 × 20 kg in each hand (Figures 2A,B); the load was chosen based on a ten-repetition maximum test performed the day before. During the ISQ performance, the knee angle ranged from 0° to 90°, while the torso-femur angle remained constant at 0° throughout the movement. The squat was performed with precise timing for all volunteers: 1 s going down (i.e., from a knee angle of 0°–90°) and 1 s coming up (i.e., from a knee angle of 90°–0°); a metronome kept time. All subjects were familiarized with the ISQ during a single training session, 4–5 days prior to the experimental testing.

### 2.4 Measurement protocol

Tensiomyography (TMG) was used to assess the contractile response of the rectus femoris (RF) muscle before and after the conditioning intervention (García-Manso et al., 2011; Đorđević et al., 2022). All measurements were conducted using a TMG S1 system (TMG-BMC, Ljubljana, Slovenia) with a sensitive digital displacement sensor. This inductive sensor incorporated a spring with a constant of 0.17 N/mm, providing an initial pressure of approximately 1.5 × 10^−2 N/mm^2 over a contact tip area of 11.3 mm^2 (Valenčič and Knez, 1997; Dahmane et al., 2001). The sensor was oriented perpendicular to the muscle belly at the thickest part of the RF, and its position was identified by palpation and marked on the skin to ensure identical placement in the post-conditioning test. Participants laid supine during measurements, with the tested leg secured at a consistent position (approximately 30° knee flexion and neutral hip angle) to standardize muscle length (Dahmane et al., 2005).

Self-adhesive surface electrodes (UltraStim^®^ Wire, Axelgaard, USA) were placed symmetrically on the RF around the sensor (Figure 4). The positive electrode (anode) was placed proximally towards the hip, and the negative electrode (cathode) distally towards the knee, each about 2–5 cm from the sensor over the muscle belly (Dahmane et al., 2001; Dahmane et al., 2005). A single electrical stimulus (1 m monophasic DC pulse) was delivered *via* the electrodes to evoke an isometric twitch contraction of the muscle. To elicit a maximal (supramaximal) response, we incrementally increased the stimulation current in small steps (e.g., 5–10 mA) with approximately 10-s pauses between stimuli until peak muscle displacement (Dm) plateaued, as outlined by Valenčič and Knez (1997) and Dahmane et al. (2001). The stimulation intensity at which this plateau occurred was taken as the supramaximal level, and all measurements were made at or just above this intensity to ensure maximal muscle activation without discomfort.

The TMG sensor recorded the radial displacement of the muscle belly in response to each stimulus at a sampling rate of 1 kHz with high spatial resolution. The TMG S1 system software was used for data acquisition and to calculate the contraction parameters from the displacement–time curve (García-Manso et al., 2011; Đorđević et al., 2022). The device was calibrated according to the manufacturer’s instructions before each session to maintain measurement accuracy. Previous studies have demonstrated that the key TMG parameters, such as contraction time (Tc) and maximal displacement (Dm), are highly reproducible and reliable under these measurement conditions (Dahmane et al., 2001; Dahmane et al., 2005). Therefore, the described TMG protocol was expected to provide consistent and objective measurements of RF muscle mechanical responses pre- and post-conditioning.

### 2.5 Estimated load on the knee joint torque

An inverse dynamics analysis of a three-segment planar human body model, assuming a quasi-static condition (see Supplementary Appendix 1) (Yoshioka et al., 2007; Fry et al., 2003; Hof, 1992), was used to estimate the load on the quadriceps muscles (especially the RF, which is loaded at the full amplitude of knee movement, 0–90 deg) during the ISQ. The model’s body segment properties were estimated using anthropometric data and the subject’s body height and body mass as input parameters (Winter 2009). The subjects’ motion in the sagittal plane was recorded on video, and one exercise repetition was selected for estimating the inter-segmental angles (Supplementary Figure S2, see Appendix). The peak value of the net knee joint torque was estimated at the lowest body position in the ISQ and normalized to the subject’s body mass to reduce the effect of inter-subject variance (Wannop et al., 2012; Moisio et al., 2003). The normalized peak values of the knee joint moment were used to compare the loading of the knee extensor muscles during the ISQ between the subjects.

The approach described above was selected for several reasons. First, inverse dynamics is a well-established method in biomechanics for estimating joint torques from kinematic data, as evidenced by studies such as Yoshioka et al. (2007). Second, video analysis provides a practical and non-invasive means to capture the necessary motion data. Third, the quasi-static assumption is appropriate, given the slow nature of the squat movements. Finally, normalizing the peak torque to body mass allows for meaningful comparisons across subjects by accounting for differences in body size.

### 2.6 Variables

**FIGURE 3:**
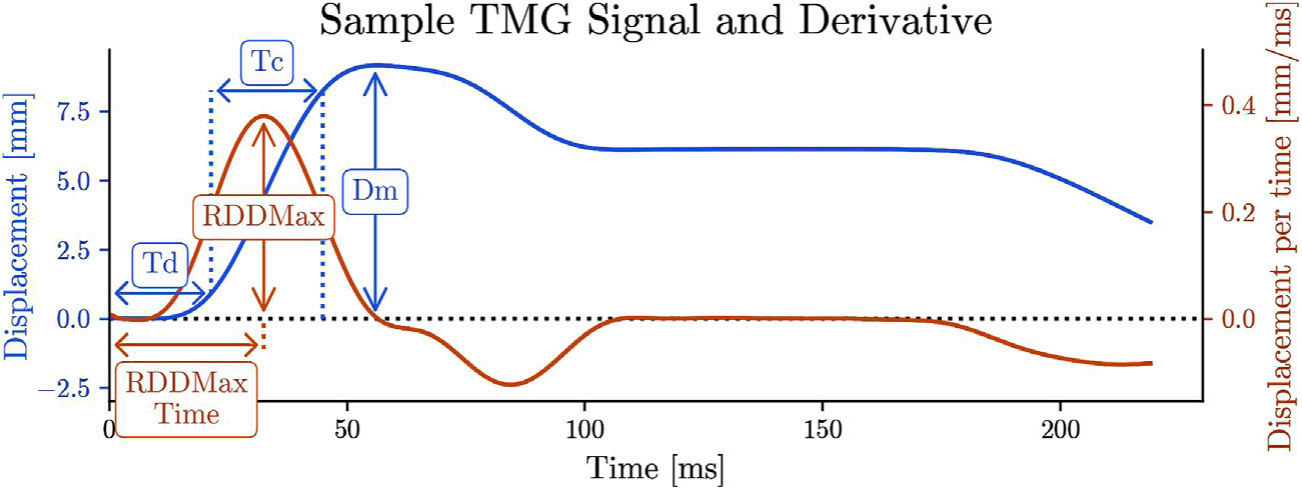
TMG signal and the discrete twitch contraction parameters. The time derivative of TMG signal. RDD stands for rate of displacement development, RDDmax, TRDDmax.

The following discrete twitch contraction parameters were computed from each TMG signal Figure 3:

• **Dm** [mm]: Maximum displacement of the muscle belly
• **Td [ms] (Delay Time)**: Time to reach 10% of Dm
• **Tc [ms] (Contraction Time)**: Time taken to change from 10% to 90% of Dm
• **RDD**_**max**_ **[mm/ms] (Maximum Rate of Displacement Development)**: Maximum value of the TMG derivative (computed using a second-order finite difference scheme)
• **TRDD**_**max**_ **[ms]**: Time of occurrence of RDD_max_ **[ms]**

The following metrics were used in the SPM analysis:

• t*: The threshold t-value determining statistical significance for spatial or temporal data.
• **T1 and T2 [ms]**: Time parameters of the onset and offset of the supra-threshold region, respectively.
• **t**_**max**_ Maximum value of the SPM t-continuum.
• **TPL (Total Potentiation Level)**: Area of the supra-threshold cluster in the (time, SPM-t) plane, calculated by the trapezoid method for numerical integration.
• **T**_**max**_ **[ms]**: Time of maximum SPM t-continuum.
• α is the significance level used for SPM t-tests, p is the associated p-value, and SD is the standard deviation

### 2.7 Statistical analysis and software

The Python three programming language (https://www.python.org/) was used for SPM analysis and TPL calculations, utilizing the Numpy and SciPy libraries (https://numpy.org/, https://scipy.org/). SPSS Statistics 20 (SPSS Inc., Chicago, IL, USA) and Microsoft Excel (Microsoft Corporation, Redmond, WA, USA) were applied for the remaining analyses, including normality tests, Student’s t-test, and standard deviation (SD) calculations. Statistical parametric mapping analysis was performed in Python three using the SPM1d library (Pataky, 2012) https://www.spm1d.org. Cohen’s (1988) framework was employed for sample size considerations and statistical power, expressing effect sizes as standardized mean differences (Cohen’s dz) for paired-sample comparisons. Subsequently, statistical power (1 – β) was calculated *post hoc* using GPower SW (GPower 3.1.9.7, Heinrich Heine University Düsseldorf, Düsseldorf, Germany) based on the observed effect sizes derived from these comparisons. The β represents the probability of a Type II error, indicating the likelihood that a study fails to detect a statistically significant difference when one genuinely exists.

The RF’s contractile response was measured with TMG, where the signal was sampled for 1s at 1 kHz for 1,000 data points. To quantify the magnitude of the twitch potentiation in the first 100 m, we calculated the area of the difference between the (before and after ISQ) time series as the integral over the statistically significant threshold value.

The TMG derivative was computed with a finite difference scheme using Numpy’s gradient function with a time-step of 1 m; the gradient function used second-order central differences and second-order one-sided forward/backward differences at the endpoints.

The Kolmogorov-Smirnov normality test was used to check whether the variables were normally distributed. The significance of differences between the defined discrete TMG parameters was evaluated using a two-tailed Student’s t-test for paired samples. Statistical Parametric Mapping (SPM) is a method that extends traditional zero-dimensional statistical tests to one-dimensional data by evaluating the probability that the maximal statistic exceeds a threshold over a smooth random field using Random Field Theory (RFT; Pataky, 2012; Pataky et al., 2015; Pataky et al., 2016). Our SPM analysis employs one-dimensional paired t-tests to examine the influence of incline squat (ISQ) conditioning on the rectus femoris muscle’s twitch contraction properties based on tensiomyography (TMG) signals recorded pre- and post-conditioning. This analysis focuses on the initial 100 m of the TMG response to highlight the contraction phase, providing a time-specific assessment of muscle response to conditioning. By comparing the pre- and post-conditioning TMG time series, areas of statistically significant differences were calculated to quantify potentiation. The SPM approach identifies these changes across the entire temporal profile, offering a comprehensive view of contraction dynamics.

Using a two-tailed SPM{t} inference at α = 0.01, we set a threshold t-statistic (t*) via RFT to control the family-wise error rate over the full time continuum by accounting for field smoothness and degrees of freedom (Adler and Taylor, 2007; Pataky, 2012). RFT uses the expected Euler characteristic of excursion sets to approximate the chance that a smooth null field would yield a supra-threshold cluster as large as observed; clusters above t* are therefore deemed significant after multiplicity correction (Adler and Taylor, 2007; Worsley et al., 1996). Assumptions we verified were approximately Gaussian residuals, weak stationarity, and sufficient temporal smoothness (Pataky, Vanrenterghem, and Robinson, 2015). We then applied a 1D paired-t SPM and integrated supra-threshold regions to compute TPL (Pataky, 2012). Statistically significant differences in the t-continuum indicate enhanced neuromuscular responses following ISQ conditioning.

## 3 Results

Descriptive statistical results of RF TMG twitch measurements before and after ISQ are presented in Table 1 as mean ± SD and as a percentage of differences before and after ISQ. When comparing the RF twitches before and after ISQ in all four series, statistically significant differences (p < 0.0001) were found for all compared parameters.

**TABLE 1:** Comparison of pre- and post-ISQ twitch contraction parameter values. Each set’s post-ISQ values are compared to the baseline pre-ISQ values fromset 1.

Set 1	^*µ*^pre	^*µ*^post	% Change	^*σ*^pre	^*σ*^post	*p*
Dm [mm] Td [ms] Tc [ms] RDD_max_ [mm ms^*−*1^] TRDD_max_ [ms]	8.66 25.2 31.3 0.27 41.2	9.96 22.4 26.0 0.37 36.6	+15% *−*11% *−*17% +39% *−*11%	2.51 2.4 4.8 0.08 6.9	2.77 1.6 3.9 0.10 5.2	3 *×* 10^*−8*^ 1 *×* 10^*−19*^ 1 *×* 10^*−20*^ 1 *×* 10^*−20*^ 5 *×* 10^*−8*^

Set 2	^*µ*^pre	^*µ*^post	% change	^*σ*^pre	^*σ*^post	*p*
Dm [mm] Td [ms] Tc [ms] RDD_max_ [mm ms^*−*1^] TRDD_max_ [ms]	8.66 25.2 31.3 0.27 41.2	10.05 22.1 25.2 0.39 35.4	+16% *−*12% *−*19% +46% *−*14%	2.51 2.4 4.8 0.08 6.9	2.64 1.6 3.9 0.10 4.8	2 *×* 10^*−10*^ 4 *×* 10^*−19*^ 1 *×* 10^*−27*^ 2 *×* 10^*−24*^ 8 *×* 10^*−12*^

Set 3	^*µ*^pre	^*µ*^post	% change	^*σ*^pre	^*σ*^post	*p*
Dm [mm] Td [ms] Tc [ms] RDD_max_ [mm ms^*−*1^] TRDD_max_ [ms]	8.66 25.2 31.3 0.27 41.2	10.16 21.9 24.9 0.40 35.3	+17% *−*13% *−*20% +49% *−*14%	2.51 2.4 4.8 0.08 6.9	2.54 1.6 4.2 0.08 4.9	2 *×* 10^*−9*^ 2 *×* 10^*−19*^ 2 *×* 10^*−26*^ 5 *×* 10^*−25*^ 9 *×* 10^*−13*^

Set 4	^*µ*^pre	^*µ*^post	% change	^*σ*^pre	^*σ*^post	*p*
Dm [mm] Td [ms] Tc [ms] RDD_max_ [mm ms^*−*1^] TRDD_max_ [ms]	8.66 25.2 31.3 0.27 41.2	9.93 21.8 24.6 0.39 35.4	+15% *−*14% *−*21% +46% *−*14%	2.51 2.4 4.8 0.08 6.9	2.45 1.9 3.7 0.08 5.4	1 *×* 10^*−6*^ 2 *×* 10^*−21*^ 7 *×* 10^*−27*^ 6 *×* 10^*−21*^ 3 *×* 10^*−13*^

Note the consistent, potentiation-like increase in muscle amplitude and decrease in contraction time following ISQ., columns are, respectively; pre-ISQ, mean value *µ*_pre_; post-ISQ, mean value *µ*_post_; percent change between pre-ISQ, and post-ISQ, mean values; pre-ISQ, standard deviation *σ*_pre_; post-ISQ, standard deviation *σ*_post_; t-test *p*-value *p*.

All four TMG parameters showed very large paired effect sizes, and the observed power was essentially 100% (1–β ≈ 1.00) for detecting the pre/post differences with N = 58. The Cohen’s dz values for Dm ranged from ∼0.71 to 1.02 across the four sets (mean dz ≈ 0.88), for Td from ∼1.71 to 1.93 (mean ≈1.78), for Tc from ∼1.94 to 2.71 (mean ≈2.45), and for RDD_max_ from ∼1.75 to 2.41 (mean ≈2.04). Given these large effects, the sample size required to achieve 90% power was very low for all parameters: on average, only about N ≈ 17 participants for Dm (range = 13–23); N = 6 for Td (range = 6–6); N = 5 for Tc (range = 4–6); and N = 6 for RDD_max_ (range = 5–6) (see tables above). In other words, the sample of 58 was far more than sufficient, yielding post-hoc power >99.9% in all comparisons. This analysis confirms that the study was highly powered to detect the observed changes in TMG parameters across all four ISQ sets.

The estimated bilateral knee muscle load, the mean value of the net knee torque at ISQ, was 275.4 ± 53.4 Nm. The torque normalized to the body and added mass was 1.395 ± 0.158 Nm/kg, indicating comparable between-subject RF muscle effort while performing the ISQ.

When examining the complete twitch response of the RF muscle before and after each ISQ, SPM using RFT analysis indicated a statistically significant difference in the total muscle twitch within the first 100 milliseconds across all four sets (see Table 2; Figures 5A–D). The SPM analysis (see Table 2) revealed a highly significant difference when comparing the rectus femoris muscle twitch response before the 1st set of ISQ with the responses measured after the 1st, 2nd, 3rd, and 4th sets of ISQ. The SPM parameters in Table 2 are derived from a one-dimensional paired two-tailed SPM{t} analysis on the initial 100 m of TMG twitch responses, capturing the temporal dynamics of potentiation. Specifically, t^∗^ denotes the significance threshold, T1/T2 defines the duration of significant changes, t_max_ and T_max_ indicate peak potentiation, and TPL integrates these to quantify overall PAP effects (as detailed in Section 2.6). The dynamic course of these differences is clearly illustrated in Figures 5A–D. Notably, the maximum magnitude of the difference (t-max = 24.35) was observed after the 2nd set, and the highest overall level of potentiation (TPL = 636.5) was also recorded following the 2nd set (Figure 5B). The onset of potentiation, defined by T1, and the offset of the supra-threshold region, defined by T2, did not vary significantly across trials.

**TABLE 2:** SPM values for comparisons of rectus femoris twitch responses pre- and post-ISQ across four sets.

ISQ set	_*t*_∗	*p*	*T*_1_ [ms]	*T*_2_ [ms]	*t*_max_	*T*_max_ [ms]	TPL
Set 1 Set 2 Set 3 Set 4	3.68 3.66 3.67 3.69	_*<* 10_–16 _*<* 10_–16 _*<* 10_–16 _*<* 10_–16	11.3 12.4 11.7 11.9	62.6 62.0 61.5 60.5	19.3 24.4 21.6 21.9	39.5 41.1 38.9 39.2	557 637 607 583

Parameters include: t^∗^ (threshold t-value for statistical significance at α = 0.01, indicating the critical value above which differences are deemed significant); p (associated p-value for the supra-threshold cluster, reflecting overall statistical significance); T1 and T2 [ms] (onset and offset times of the supra-threshold region in the SPM t-continuum, defining the temporal window of significant potentiation); t_max_ (maximum value of the SPM t-continuum, representing the peak magnitude of difference); T_max_ [ms] (time at which t_max_ occurs, indicating the point of maximal potentiation within the twitch response); and TPL, calculated as the integrated area of the supra-threshold cluster in the (time, SPM-t) plane using the trapezoid method, providing a holistic measure of potentiation magnitude and duration). All analyses were conducted with α = 0.01 significance level.

The shape of the SPM t-continuum differed between sets, with the most pronounced deviation observed when comparing the initial measurement to the measurement following the 2nd set of ISQ (Table 2).

Additionally, one-dimensional paired SPM contrasts of baseline pre-set twitches (Pre1 vs. Pre2, Pre1 vs. Pre3, and Pre1 vs. Pre4), using RFT-based thresholding over 0–100 m at α = 0.01, revealed no supra-threshold clusters; thus, there was no statistically significant evidence of cumulative peripheral fatigue across sets.

## 4 Discussion

This study investigated the effects of PAP using TMG and SPM to quantify and characterize changes in muscle twitch responses. These methodologies comprehensively assessed muscle potentiation, allowing for a detailed capture of its magnitude and temporal dynamics in response to a conditioning stimulus.

**FIGURE 4:**
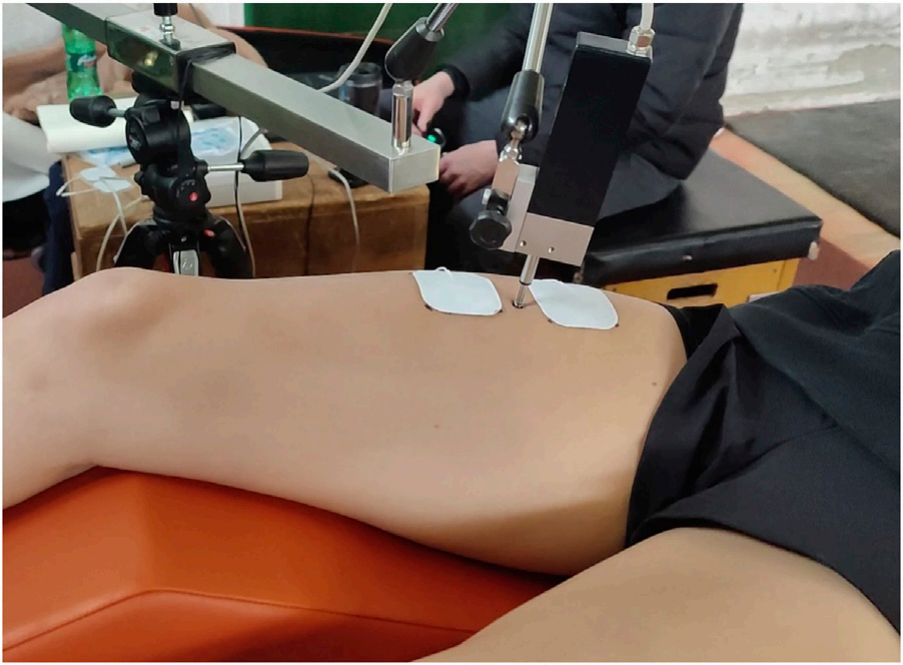
The position of the electrodes and sensor tip during electrical stimulation.

**FIGURE 5:**
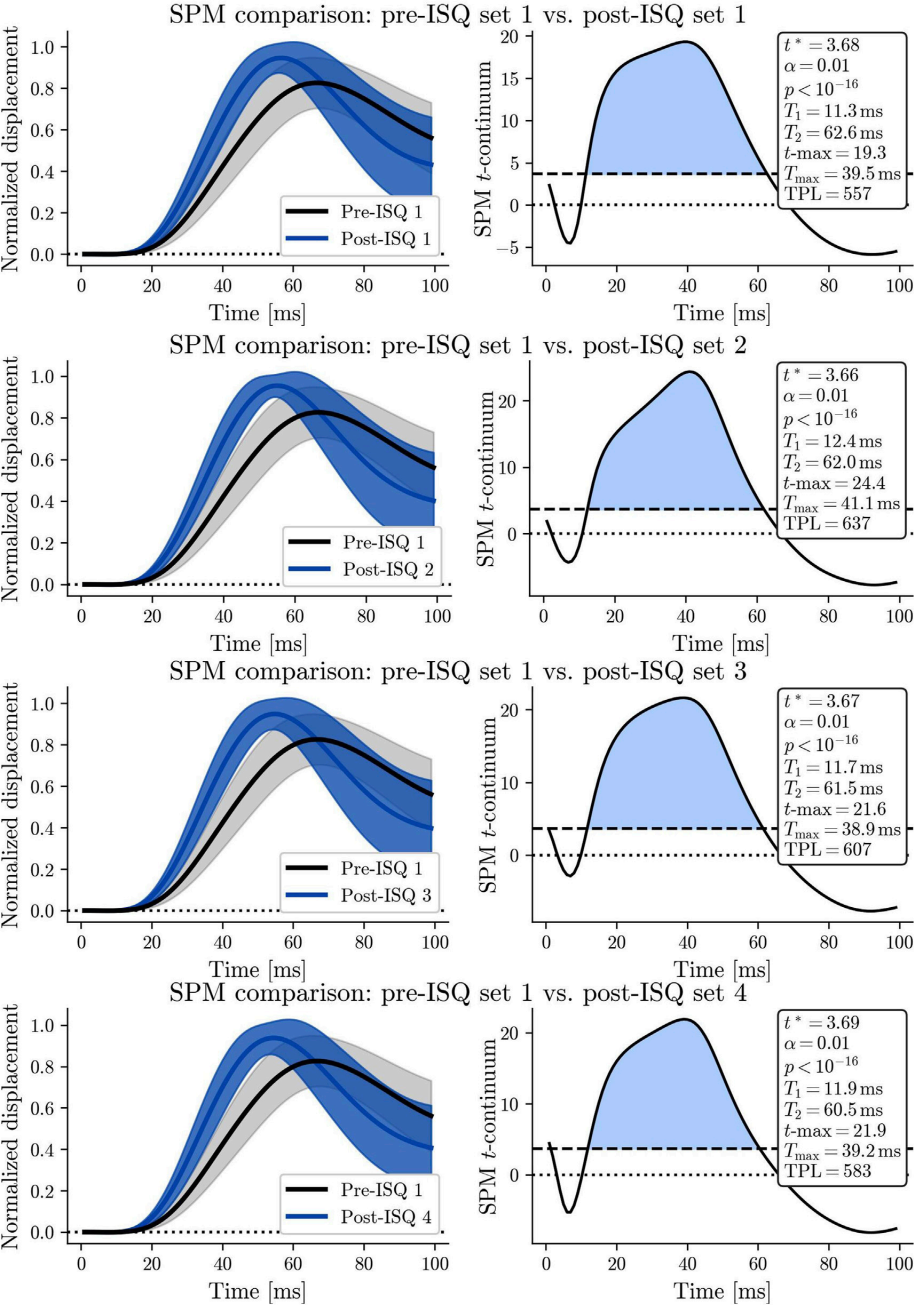
**(A)** The SPM plot on the right illustrates the time course of the rectus femoris muscle twitch response for the first set pre-ISQ (black line) and the first set post-ISQ (blue line), with both lines representing the mean of all measurements. The shaded areas surrounding each line indicate the confidence intervals, reflecting the variability in the data. On the left, the SPM t-continuum highlights the specific time points where statistically significant differences between the two conditions are observed. **(B)** The SPM plot on the right illustrates the rectus femoris muscle twitch response time for the first set pre-ISQ (black line) and the second set post-ISQ (blue line), with both lines representing the mean of all measurements. **(C)** The SPM plot on the right illustrates the time course of the rectus femoris muscle twitch response for the first set pre-ISQ (black line) and the third set post-ISQ (blue line), with both lines representing the mean of all measurements **(D)** The SPM plot on the right illustrates the rectus femoris muscle twitch response time for the first set pre-ISQ (black line) and the fourth set post-ISQ (blue line), with both lines representing the mean of all measurements.

The SPM analysis of the muscle twitch (Table 2; Figures 5A–D) revealed an apparent increase in potentiation from the first to the second set of ISQ, reaching its peak before declining in the third and fourth sets. Interestingly, this dynamic pattern was not reflected in the discrete statistical analysis (Table 1), as none of the measured discrete variables—such as contraction time (Tc), delay time (Td), or rate of force development (RDD_max_)—exhibited similar trends. This discrepancy highlights the sensitivity of SPM (Pataky,2012) in detecting subtle continuous changes in the muscle twitch that may be missed by discrete statistical analysis (Hughes et al., 2022). While discrete analysis focuses on isolated parameters at specific points within the twitch response, potentially overlooking the gradual build-up and decay of potentiation, SPM analyzes the entire time course of the muscle twitch response, detecting subtle shifts in potentiation throughout the contraction Figure 4. This integrated approach provides a more comprehensive understanding of muscle’s adaptive responses. Practically, repeated PAP induction verified by TP-derived metrics (e.g., TPL) is intended to inform the progressive individualization of explosive-strength training—targeting rapid high-threshold motor-unit recruitment and early rate of force development—i.e., a chronic, training-driven application rather than an acute performance test. In parallel, long-term improvements in core stability and neuromuscular coordination—shown to increase lower-limb peak torque and reduce inter-limb asymmetry—may augment the potentiation effects detected by TPL, indicating a plausible synergy between acute PAP responses and chronic training adaptations (Dello Iacono et al., 2016).

Although PAP and PAPE are interconnected, their relationship is intricate, and the presence of PAP does not necessarily lead to PAPE. Conceptually, PAP has two manifestations: an acute, short-lived effect (often discussed as PAPE) and a chronic, training-relevant effect that emerges from the repeated induction of PAP across sessions. Nevertheless, when PAP reaches a sufficiently high level, it is more likely to be associated with PAPE. This suggests that optimizing PAP (and thus maximizing TPL) may increase the likelihood of achieving PAPE, thus potentially enhancing performance efficiency. However, in this study, our primary interest lies in the chronic dimension—using TPL as a monitoring tool to optimize conditioning prescriptions over time, rather than in quantifying immediate, task-specific PAPE. Empirical evidence suggests that greater PAP is associated with improved explosive performance. Requena et al. (2011) found that soccer players with higher PAP in the knee extensors after a 10-s maximal contraction achieved greater jump heights and faster 15-m sprint times, with jump height correlating positively (r ≈ 0.61–0.64) and 15-m sprint time correlating inversely (r = −0.59) with PAP. Similarly, Bergmann et al. (2013) reported a ∼12% increase in drop jump height (p < 0.05) following a plyometric conditioning activity (hops), which correlated with increased twitch torque (*r* ^2^ = 0.26, P < 0.05).

Detecting and analyzing potentiation is essential for revealing the muscular system’s adaptations to high-speed and explosive movements (Hodgson et al., 2005). By tailoring the intensity and specificity of conditioning stimuli, these adaptations can enhance health (Seitz and Haff, 2016; Izquierdo et al., 2021) and improve performance across various sports and physical activities (Hodgson et al., 2005; Boullosa et al., 2018; Seitz and Haff, 2016). Additionally, a correlation between TP and the activation of fast-twitch muscle fibers has been found (Sasaki et al., 2012). Using TP as a non-invasive tool to measure fast-twitch fiber activation has significant implications in medical diagnostics and sports performance monitoring, offering a novel approach to assess and enhance muscle function.

The SPM analysis showed significant differences between the rectus femoris twitch response before the first set of ISQ and the responses measured after the first, second, third, and fourth sets. These differences were most pronounced after the second set, where the maximum magnitude of potentiation (t-max = 24.35) and the highest overall potentiation level (TPL = 636.52) were observed. In contrast, traditional discrete analyses did not exhibit similar trends across sets (Table 1), such as changes in contraction time (Tc), delay time (Td), or rate of force development (RDD_max_). This discrepancy underscores the sensitivity of SPM in capturing temporal and amplitude dynamics that these discrete measures might miss. By evaluating the entire temporal profile of muscle contraction, SPM provides a richer analysis, allowing for detecting subtle yet critical changes in muscle behavior, which is particularly relevant for applications in sports performance and rehabilitation, where optimizing muscle response is critical. The TPL metric presents opportunities for integrating muscle stability training with protocols incorporating stability elements, as it sensitively quantifies PAP dynamics that may enhance neuromuscular adaptations in stability-focused exercises. Drawing Dello Iacono et al. (2016), who demonstrated the effects of core stability training on lower limb balance strength and performance outcomes without involving plyometric interventions, TPL could refine combined training regimens by monitoring potentiation thresholds to optimize stability and coordination outcomes while mitigating excessive fatigue and maximizing TPL values. TPL framework can represent a promising advancement in quantifying PAP, offering a more comprehensive metric that encompasses the total potentiation over time. Unlike traditional peak-based measures, TPL accounts for both the magnitude and the duration of potentiation, providing a richer and more informative measure of neuromuscular response. This approach helps to characterize the potentiation process better, highlighting its peak effects and temporal persistence, which could prove crucial in optimizing conditioning and rehabilitation protocols. This suggests that potentiation develops across the sets, peaking after the second set and then beginning to plateau or diminish slightly, likely due to the onset of fatigue. The onset (T1 = 11.3 m) and offset (T2 = 62.6 m) of the supra-threshold region in the SPM analysis remained relatively consistent across trials (Table 2). This consistency implies that the mechanisms responsible for PAP—such as myosin light chain phosphorylation and increased calcium sensitivity—are activated within a predictable time window following the conditioning activity.

The detection and quantification of PAP via TP are relevant for developing new methods to improve movement speed and muscle power, which are important factors in sports (Tillin and Bishop, 2009), rehabilitation, and anti-aging interventions (Lundqvist et al., 2022). PAP can also reflect the adaptive capacity of muscles and help optimize the loading protocols that induce this adaptation.

The shape of the SPM t-continuum varied across sets, indicating changes in the pattern of potentiation over time. The most pronounced deviations occurred between the initial pre-ISQ measurement and the second set post-ISQ (Figure 5B). This variation reflects how the muscle’s contractile properties adapt with each subsequent conditioning stimulus, highlighting the transient and cumulative nature of PAP. Significant changes occur after just a few conditioning sets, emphasizing that potentiation is a gradual process with noticeable effects early on.

Additionally, our findings align with those of Rassier and Macintosh (2000), illustrating the dynamic interplay between potentiation and fatigue. PAP enhances muscle performance by increasing the magnitude and speed of contraction, as evidenced by the improvements in TMG signals. However, after the second set, further conditioning does not significantly amplify potentiation, likely because the specific form of fatigue begins to counteract its benefits. This balancing act suggests that while PAP can improve performance in short bursts, there is a threshold beyond which additional conditioning may not yield further gains and might even be detrimental due to fatigue onset. Muscle fatigue can indeed have a negative impact on both the learning of new motor skills and the optimization of existing movement patterns. This effect occurs because fatigue interferes with the effective functioning of the central and peripheral components of the neuromuscular system, which are crucial for motor learning and control. Specifically, central fatigue, which impacts neural drive, and peripheral fatigue, which affects muscle force production, reduces movement accuracy and consistency, impeding motor learning (Gandevia, 2001; Torreblanca-Martínez et al., 2020).

These results underscore the importance of optimizing conditioning or rehabilitation protocols to maximize TPL while minimizing fatigue. Practitioners should consider that potentiation effects peak after a certain point—in this case, after the second set—and additional loading may not provide further benefits. Incorporating SPM into muscle function assessments offers a more sensitive tool for tracking the dynamic interplay between potentiation and fatigue, allowing for more precise adjustments to training regimens. These findings suggest that the principle of optimal potentiation can inform athletic coaches and rehabilitation therapists in determining the appropriate frequency and duration of conditioning exercises. Specifically, timing the exercises to avoid excessive conditioning beyond the second set may enhance gains while minimizing fatigue. Our findings align with previous studies that utilized TMG to assess PAP. García-Manso et al. (2011) reported increased muscle contractility following similar conditioning protocols, demonstrating that TMG effectively detects changes in muscle mechanical properties post-exercise. Similarly, Abazović et al., 2022 emphasized that twitch potentiation measured by TMG is a clear and reliable indicator of potentiation, highlighting the method’s sensitivity and direct measurement of muscle response *in situ*. Although fatigue clearly influenced PAP in our protocol, we did not attempt to partition its components (peripheral, central, or subjective), as the primary objective of this study was to introduce and validate TPL as a PAP metric. We acknowledge that this complex interplay can complicate the interpretation of our findings, representing a potential limitation of this study. It is noteworthy that TMG, as employed in the current study, primarily gauges peripheral fatigue mechanisms *via* the radial displacement of the muscle during an evoked twitch, and recent research confirms TMG’s sensitivity to exercise-induced changes in muscle contractility (Piqueras-Sanchiz et al., 2024). However, the ability of TMG-derived displacement parameters to reflect the net balance between potentiation and fatigue may differ from that of twitch torque measurements under certain conditions (e.g., specific joint angles or muscle loading patterns), potentially explaining some discrepant findings in the literature (Langen et al., 2025). Additionally, pairing TPL with brief proprioceptive and or coordination tests may help contextualize when potentiation is most likely to be expressed in voluntary tasks (Frizziero et al., 2023).

Unlike some studies that did not observe a decline in potentiation after multiple sets (Tillin and Bishop, 2009), our results indicate a slight decrease in potentiation beyond the second set. However, despite this slight decline, the level of potentiation after the second set remained higher than after the first set. These differences are likely due to variations in conditioning protocols across different studies. A slight decline is likely due to cumulative fatigue effects. Rassier and Macintosh (2000) discussed the coexistence of potentiation and fatigue in skeletal muscle, suggesting that while potentiation enhances force production, fatigue mechanisms can concurrently reduce it. Our observation of potentiation peaking after the second set, followed by a decline, aligns with their findings on the dynamic interplay between these two phenomena.

While the present study did not define fixed thresholds for TPL, our findings suggest that higher TPL values reflect greater neuromuscular engagement and could signal an increased potential for performance adaptation. TPL appears highly individual and context-dependent, varying with training status, exercise type, and individual responsiveness. Rather than a limitation, this variability may position TPL as a valuable metric for tailoring PAP protocols to individual needs. As such, TPL could serve as a practical, non-invasive tool to guide conditioning load, with higher values indicating an effective stimulus and sharp declines potentially marking the onset of fatigue-related impairments.

### 4.1 Limitations

While this study provides valuable insights into quantifying PAP using tensiomyography and SPM statistical analysis, several limitations should be acknowledged. First, the participant pool consisted of healthy, physically active individuals, which may limit the generalizability of the findings to other populations, such as sedentary individuals, older adults, or those with neuromuscular disorders. Second, the study focused exclusively on the rectus femoris muscle using a specific conditioning exercise - the weighted incline squat. Therefore, the results may not directly apply to other muscles or conditioning modalities. Third, we assessed the immediate effects of PAP without evaluating long-term adaptations or the sustainability of potentiation effects over time. Potential confounding factors such as individual differences in muscle fiber composition, prior training status, and fatigue levels were not extensively controlled or examined.

Future research should address this gap by integrating TMG/TPL analysis with methods that assess central and subjective fatigue, thereby providing a more comprehensive understanding of neuromuscular responses to conditioning exercises.

## 5 Conclusion

The combination of TMG and SPM has introduced a new metric, TPL, which quantifies the magnitude of PAP with high sensitivity. By capturing changes in both amplitude and temporal dimensions of muscle contraction, TPL provides a comprehensive understanding of PAP dynamics, distinguishing potentiation from concurrent fatigue.

Our analysis detected statistically significant initial changes in the TMG signal of the RF muscle just after 11.3 m, persisting up to 62.6 m. These findings suggest that potentiation effects span much of the signal’s rising phase. Changes in both temporal and amplitude components across four conditioning sets further imply the potential of this methodology to define distinct types of PAP. The practical utility of TMG and SPM makes them valuable tools for evaluating muscle function and refining conditioning protocols in scientific research, rehabilitation, athletics, and aging populations.

In conclusion, the present study introduces the TPL as a novel metric for quantifying the dynamics of post-activation potentiation (PAP) using combined tensiomyography (TMG) and one-dimensional statistical parametric mapping (SPM). The underlying framework can be extended to additional muscle groups and diverse populations, provided that appropriate validation is conducted. For example, future applications might examine other biarticular muscles (such as the biceps femoris of the hamstrings and the gastrocnemius) and various athletic or clinical populations to assess the generalizability of TPL.

Future research should pursue the following directions:

1. Evaluate generalizability across different target populations, muscle groups (including biarticular muscles like biceps femoris and gastrocnemius), and conditioning modalities.
2. Integrate simultaneous surface electromyography (e.g., as in Sun et al., 2022) with TMG measurements to elucidate neural contributions to PAP and fatigue.
3. Compare TPL with complementary techniques (e.g., the M-wave, which reflects motor unit activation, or direct force measurements) to refine the mechanistic interpretation of potentiation and neural activation.
4. Critically test the hypothesis that a TPL-guided PAP induction paradigm–i.e., structuring training sessions to elicit high TPL while managing fatigue consistently–yields superior long-term improvements in early-phase rate of force development (e.g., 0–50 m and 0–100 m) and in explosive performance outcomes (such as countermovement jump height, horizontal jump distance, and short-sprint times) compared to standard training protocols.

Collectively, these investigations will clarify the boundaries and scope of TPL, bolster its external validity, and position it as a practical tool in athletic training and neuromuscular performance assessment.

## Data Availability

The raw data supporting the conclusions of this article will be made available by the authors, without undue reservation.
