# Supplementary material for: Total potentiation level: a new metric for quantifying post-activation potentiation dynamics using tensiomyography and statistical parametric mapping
Source: Front Bioeng Biotechnol. 2025 Sep 9;13:1533749. doi: 10.3389/fbioe.2025.1533749 (PMC12454992; doi:10.3389/fbioe.2025.1533749)
Supplement: Supplementary file 1 [file Supplementaryfile1.docx]

Appendix

# Inverse dynamics analysis of incline squat

The inverse dynamics analysis of the incline squat is based on a planar 3-link model, where each link is assumed to be a rigid body segment: 1 – shanks, 2 – thighs, trunk, neck, and head, 3 – arms. The 3-link model, as depicted in Figure 1, represents an open kinematic chain, where the foot segment is fixed to the ground and the motion of the body segments is constrained by revolute joints: the ankle joint (between the foot and the shank), the knee joint (between the shank and the upper leg), and the shoulder joint (between the trunk and the arm). The external load – free weight, $F_{e,y}$, is applied to the most distal point of the body segment 3.

| 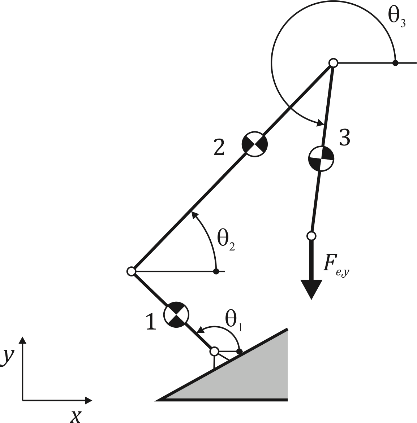 | 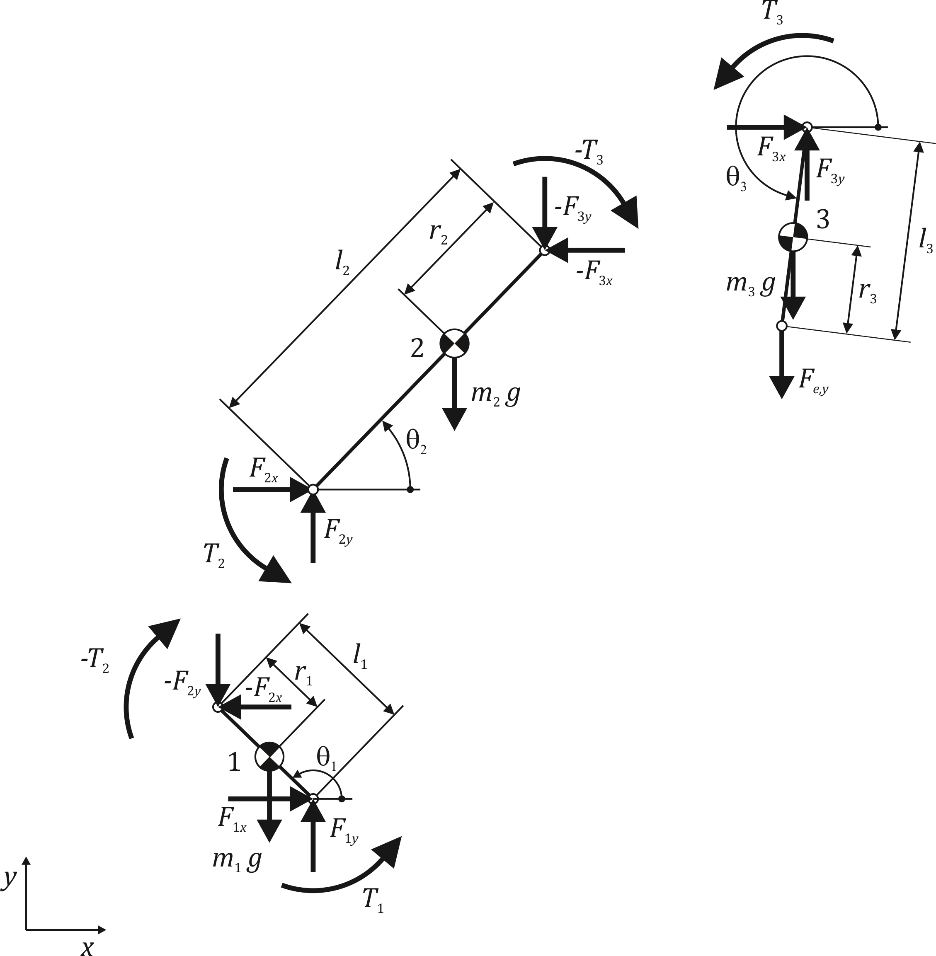 |
| --- | --- |
| Figure 1. The 3-link model of the incline squat and the free-body diagram of body segments and reactions. | |

Three equations can be written for each body segment, representing the summation of externally applied and reaction forces and torques. The dynamic equilibrium of the three body segments can be described by three equations, representing the summation of externally applied and reaction forces and torques. The planar dynamics of the 3-link model is described by a system of nine equations that can be solved for the reaction forces $F_{1x}$, $F_{1y}$, $F_{2x}$, $F_{2y}$, $F_{3x}$, $F_{3y}$ and joint torques $T_{1}$, $T_{2}$, $T_{3}$ needed for dynamic equilibrium.

Body segment 1 (left shank, right shank):

$$F_{1x}-F_{2x}=m_{1}a_{1x}$$

$$F_{1y}-F_{2y}-m_{1}g=m_{1}a_{1y}$$

$$F_{1x}\left( l_{1}-r_{1} \right)\sin\theta_{1}-F_{1y}\left( l_{1}-r_{1} \right)\cos\theta_{1}+T_{1}+F_{2x}r_{1}\sin\theta_{1}-F_{2y}r_{1}\cos\theta_{1}-T_{2}=J_{1}\alpha_{1}$$

Body segment 2 (trunk, neck, head, left thigh, right thigh):

$$F_{2x}-F_{3x}=m_{2}a_{2x}$$

$$F_{2y}-F_{3y}-m_{2}g=m_{2}a_{2y}$$

$$F_{2x}\left( l_{2}-r_{2} \right)\sin\theta_{2}-F_{2y}\left( l_{2}-r_{2} \right)\cos\theta_{2}+T_{2}+F_{3x}r_{2}\sin\theta_{2}-F_{3y}r_{2}\cos\theta_{2}-T_{3}=J_{2}\alpha_{2}$$

Body segment 3 (left arm, right arm):

$$F_{3x}=m_{3}a_{3x}$$

$${-F}_{e,y}+F_{3y}-m_{3}g=m_{3}a_{3y}$$

$$+F_{3x}\left( l_{3}-r_{3} \right)\sin\theta_{3}-F_{3y}\left( l_{3}-r_{3} \right)\cos\theta_{3}+T_{3}-F_{e,y}r_{3}\cos\theta_{3}=J_{3}\alpha_{3}$$

For the inverse dynamics analysis, quasi-static conditions were assumed, with the body segment translational accelerations $a_{1x}$, $a_{1y}$, $a_{2x}$, $a_{2x}$, $a_{3x}$, $a_{3x}$ and angular accelerations $\alpha_{1}$, $\alpha_{2}$, $\alpha_{3}$ equal to zero, and only gravitational acceleration $g$ acting at the center of mass. The body segment angles $\theta_{1}$, $\theta_{2}$ and $\theta_{3}$ were obtained from a video of the volunteer 2D kinematics in the sagittal plane (knee extension angle: ${180^{\circ}-\theta}_{1}+\theta_{2}$). The volunteer’s body height and mass were used to estimate the body segment properties: the length ($l_{1}$, $l_{2}$, $l_{3}$), the mass ($m_{1}$, $m_{2}$, $m_{3}$), the center of mass location ($r_{1}$, $r_{2}$, $r_{3}$) and the inertia moment ($J_{1}$, $J_{2}$, $J_{3}$).

An exemplary sequence of the volunteer’s body position is shown in Figure 2, each providing the angles between the body segments as input parameters for the 3-link model. As can be seen for the exemplary test, the knee torque $T_{2}$ increases with lowering the volunteer’s body position during the inclined squat, yielding the maximum in the lower position, representing the maximum load for the knee extensor muscles (Table 1).

| 1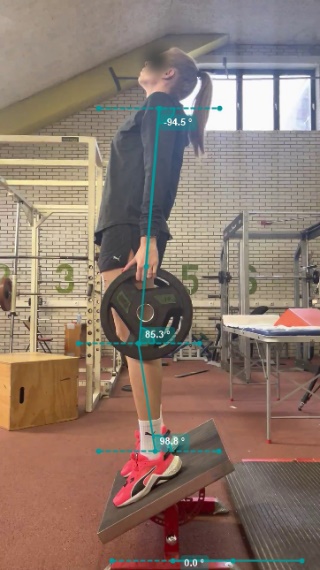 | 2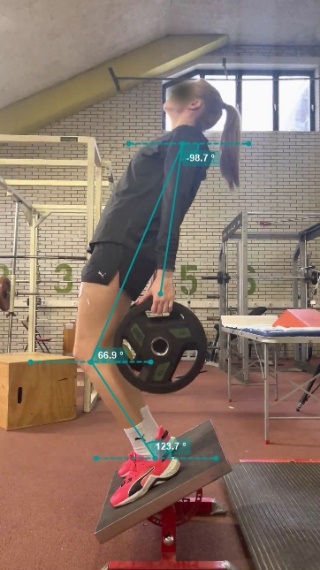 | 3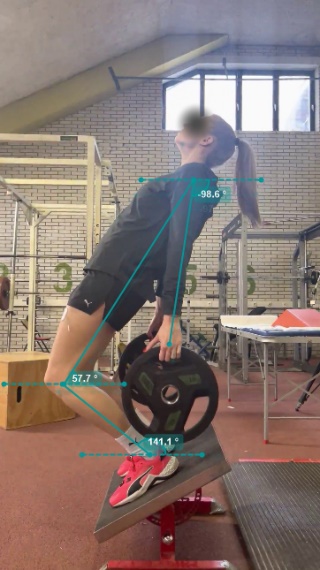 | 4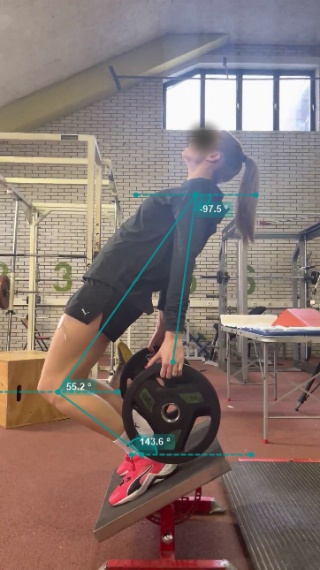 |
| --- | --- | --- | --- |
| Figure 2. An exemplary volunteer test: the angles between the body segments measured from the test video. A sequence of a sissy squat movement is shown from the upper (1) to the lower position (4). | | | |

Table 1. The angles between the body segments and the reaction moments are shown in Figure 2.

| Position | $\theta_{1} [^{\circ}]$ | $\theta_{2} [^{\circ}]$ | $\theta_{3} [^{\circ}]$ | $knee ext. [^{\circ}]$ | $T_{1} [\mathrm{Nm}]$ | $T_{2} [\mathrm{Nm}]$ | $T_{3} [\mathrm{Nm}]$ |
| --- | --- | --- | --- | --- | --- | --- | --- |
| 1 | 98.8° | 85.3 | -94.5 | 166.5 | -27.25 | 25.06 | -14.19 |
| 2 | 123.7° | 66.9 | -98.7 | 123.2 | -29.14 | 160.58 | -27.36 |
| 3 | 141.1° | 57.7 | -98.6 | 96.6 | -37.18 | 228.92 | -27.05 |
| 4 | 143.6° | 55.2 | -97.5 | 91.6 | -25.44 | 249.77 | -23.61 |
